# Supplementary material for: Whole blood transcriptomic analysis of beef cattle at arrival identifies potential predictive molecules and mechanisms that indicate animals that naturally resist bovine respiratory disease
Source: PLoS One. 2020 Jan 13;15(1):e0227507. doi: 10.1371/journal.pone.0227507 (PMC6957175; doi:10.1371/journal.pone.0227507)
Supplement: Attachment — Submitted filename: Scott et al 2019.docx [file pone.0227507.s005.docx]

Review on manuscript by Scott et al.

Whole blood transcriptomic analysis of beef cattle …

In this manuscript, the authors reported their study to examine and identify differentiated expressed genes in cattle which may play a role in resistance to BRD. They collected blood samples from cattle on arrival, subsequently, they used a set of criteria to identify those animals that are healthy or had BRD, and performed whole transcriptome analysis of their blood samples. The data was then extensively analysed by several platforms to identify differentially expressed genes in the healthy animals which may contribute to their resistance to BRD.

This is a preliminary study to identify potential host factors/immune pathways that could be involved in resistance to BRD. The study was well planned, though sample size used in the study was small as recognized by the authors. The technical part of the transcriptome analysis and date analysis was extensively and well carried out. However, the authors tried to make too much of the findings regarding the role of the DFGs. In this first study, caution should be taken with such small sample size. I have the following comments:

1. It has been well established that bacterial pathogens (and viral pathogens to a minor degree) are the cause of BRD. Even though this study is aimed at the host factors, the authors made no mention of the bacterial pathogens. E.g. What pathogens were recovered from the the animals at necropsy?
2. Along the same line, I suggest the author should consider examining the lung tissue/fluid from animals, both healthy and diseased from BRD. Total transcriptome analysis of DEGs from this location should provide more evidence to host genes and proteins involved in resistance to BRD.
3. They identified five pathways which were over expressed in healthy animals, are there any genes/pathways that are under expressed in the BRD animals? They should look into the lack of an immune response which may contribute to colonization and proliferation of bacterial pathogens in BRD.
4. For a more complete study, additional blood samples should be taken, perhaps weekly, to provide a trend in gene expression, currently, the analysis is based on one data point per animal.
