# Supplementary material for: Whole blood transcriptomic analysis of beef cattle at arrival identifies potential predictive molecules and mechanisms that indicate animals that naturally resist bovine respiratory disease
Source: PLoS One. 2020 Jan 13;15(1):e0227507. doi: 10.1371/journal.pone.0227507 (PMC6957175; doi:10.1371/journal.pone.0227507)
Supplement: Attachment — Submitted filename: Response to Reviewers.docx [file pone.0227507.s006.docx]

Mississippi State University

College of Veterinary Medicine

P.O. Box 6100

240 Wise Center Drive

Mississippi State, MS 39762

To the Editor,

We thank the reviewers for their comments regarding our manuscript (PONE-D-19-24105). We have addressed the comments and have made revisions accordingly. Our responses have been addressed in a bullet point manner below, and all changes are conveyed through Microsoft Word Track Changes in the “Revised Manuscript with Track Changes” document. Line numbers indicated in our responses below refer to the version of the revised manuscript without changes tracked.

Again, we thank you for your time and effort made and hope that our revised version is now acceptable for publication in PLOS ONE.

Sincerely,

Dr. Matthew Scott

On behalf of all authors.

**Response to Reviewer 1:**

Reviewer #1: Review on manuscript by Scott et al.

Whole blood transcriptomic analysis of beef cattle …

In this manuscript, the authors reported their study to examine and identify differentiated expressed genes in cattle which may play a role in resistance to BRD. They collected blood samples from cattle on arrival, subsequently, they used a set of criteria to identify those animals that are healthy or had BRD, and performed whole transcriptome analysis of their blood samples. The data was then extensively analyzed by several platforms to identify differentially expressed genes in the healthy animals which may contribute to their resistance to BRD.

This is a preliminary study to identify potential host factors/immune pathways that could be involved in resistance to BRD. The study was well planned, though sample size used in the study was small as recognized by the authors. The technical part of the transcriptome analysis and date analysis was extensively and well carried out. However, the authors tried to make too much of the findings regarding the role of the DFGs. In this first study, caution should be taken with such small sample size. I have the following comments:

1. *It has been well established that bacterial pathogens (and viral pathogens to a minor degree) are the cause of BRD. Even though this study is aimed at the host factors, the authors made no mention of the bacterial pathogens. E.g. What pathogens were recovered from the animals at necropsy?*

- On revised manuscript lines 140-142, we included bacterial lung culture results from the 3 animals at necropsy: all 3 animals were lung culture positive for *Mannheimia haemolytica*.

2. *Along the same line, I suggest the author should consider examining the lung tissue/fluid from animals, both healthy and diseased from BRD. Total transcriptome analysis of DEGs from this location should provide more evidence to host genes and proteins involved in resistance to BRD.*

- On revised manuscript lines 509-511, we have added the comment that future experiments involving a more total transcriptomic analysis would be beneficial in further evaluating and validating potential molecular biomarkers for animals that both resist and acquire BRD.

3. *They identified five pathways which were over expressed in healthy animals, are there any genes/pathways that are under expressed in the BRD animals? They should look into the lack of an immune response which may contribute to colonization and proliferation of bacterial pathogens in BRD.*

- On lines 398 - 402 of the revised manuscript we have noted: “Two genes involved with SPM production were lower in expression in animals that developed BRD, relative to animals that remained healthy: ALOX15 and HPGD. The gene ALOX15 encodes arachidonate 15-lipoxygenase, an enzyme that is expressed in macrophages, neutrophils, and airway epithelial cells, and has been shown to directly regulate inflammation, innate immunity, and epithelial wound healing…”. Thus, these are among the genes that were under expressed in BRD animals. While our findings cannot confirm the lack of a specific immune response to bacterial pathogens in animals that are later diagnosed with clinical BRD, our results indicate that 1) mRNA for immunologically-stimulated molecules are being expressed at arrival in both groups and 2) the lack of expression of genes in pathways related to SPM production suggests a critical role for these pathways in disease.

4. *For a more complete study, additional blood samples should be taken, perhaps weekly, to provide a trend in gene expression, currently, the analysis is based on one data point per animal.*

- We agree and appreciate that an experiment including serial sampling could yield additional valuable information about gene expression trends over time in relation to disease status.

In lines 386-388 of the revised manuscript we note that serial sampling and transcriptomic analysis could better define gene expression relationships over time with the DEGs/pathways of interest.

**Response to Reviewer 2:**

Reviewer #2: The manuscript “Whole blood transcriptomic analysis of beef cattle at arrival identifies potential predictive molecules and mechanisms that indicate animals that naturally resist bovine respiratory disease”

The authors reported the results of RNAseq data regarding the transcriptome of beef cattle that develop BRD compared to beef cattle that don’t develop the disease. In general, the manuscript is well written and rise some interesting novelties to a better understanding of the BRD.

Some of my comment can help to improve the manuscript.

*In the abstract are missing information about the experimental plan. Please add a brief description of your study.*

- Information about the number of animals, toolkits, and downstream analysis software was added to the abstract.

*Line 116-137: I find this part difficult to follow, try to simplify that*.

- Text in this section (now lines 119 - 144 of the revised manuscript) regarding animal enrollment, especially regarding vaccination status, has been modified in an effort to simplify.

*Line 134. How did you make the samples selection?*

- Added detail on lines 135-137 of the revised manuscript, emphasizing the even distribution of vaccination status at arrival as a basis of sample selection.

*Line 260-263: it is obvious that using the 36 DEG you have success in the clustering analysis.*

- We agree and appreciate the comment from the reviewer.

*Line 306, 322, 336, 377 …. the use of increased or decreased in the contest of your study is not correct. Instead, you have a higher/lower expression of the gene. Please correct throughout the manuscript.*

- Changed increased/decreased to higher/lower in expression throughout the manuscript

*Line 363-375: in this paragraph you correctly described some limitation of your study mainly about sample size. For my experience the sample number of your study is fine. The real limitation of your study is that you only analyze transcriptome. You find some interesting metabolic pathway but without metabolic confirmation. Please add a clear statement about this aspect as limitation of your study.*

- We have addressed this in lines 383-386 of the revised manuscript. We agree and appreciate the value that metabolomic or proteomic analysis could have had on confirming/validating the presence and activity of particular pathways directed by these DEGs, and hope to be able to make these assessments in future research.

*Line 418-444: this part must be reduced. Consider also that it is out of contest because you are working with Whole blood cells not with liver!*

- This section (now lines 441 - 454 in the revised manuscript) has been edited and shortened

*Line 459: what do you mean with “unchecked”?*

- Now line 469 in the revised manuscript; replaced “unchecked” with “persistent” to remove ambiguity

*CONCLUSION: specified more clearly that your study is related to circulating leucocytes*

- Language in lines 503 - 504 of the revised manuscript was included to indicate that several DEGs represent leukocytic processes

*Figure 1. change disease to BRD*

- We believed the reviewer is referring to Figure 2; the term “disease” was changed to “BRD”

*Figure5 insert in the figure or in the caption that il6 and TLR4 are only predicted in your study.*

- Figure 5 caption (lines 330-334 of the revised manuscript) were modified to specifically indicate that TLR-4 and IL-6 were not DEGs in this dataset, but the DEGs found to be higher in expression in diseased animals possessed predicted interactions with them. Lines 487 - 490 further indicate that TLR-4 and IL-6 were predicted interactors, not DEGs.
